# Injury surveillance in elite Paralympic athletes with limb deficiency: a retrospective analysis of upper quadrant injuries

**DOI:** 10.1186/s13102-020-00183-y

**Published:** 2020-06-11

**Authors:** N. R. Heneghan, L. Heathcote, P. Martin, S. Spencer, A. Rushton

**Affiliations:** 1 Centre of Precision Rehabilitation for Spinal Pain (CPR Spine), School of Sport, Exercise & Rehabilitation Sciences, University of Birmingham, Edgbaston, Birmingham, B15 2TT UK; 2 The English Institute of Sport, The Manchester Institute of Health and Performance, 299 Alan Turing Way, Manchester, M11 3BS UK

**Keywords:** Amputee, Elite sport, Injury surveillance, Limb deficiency, Paralympic medicine, Shoulder injury

## Abstract

**Background:**

Compared to injury surveillance in Olympic athletes relatively little literature exists for Paralympic athletes. Injury surveillance data underpin design and evaluation of injury prevention strategies in elite sport. The aim of this study is investigate upper quadrant injuries in elite athletes with limb deficiency.

**Methods:**

A retrospective analysis of upper quadrant injuries in elite athletes with limb deficiency with available data (2008–2016) was conducted using medical notes extracted from English Institute of Sport (EIS) records. Eligibility criteria included funded athletes, eligible for EIS physiotherapy support with an upper and/or lower limb disability arising from full or partial limb deficiency.

**Results:**

A total 162 injuries from 34 athletes were included. Participant characteristics: 20 males (59%), from 9 sports, with mean age 27 years (range 16–50 years) and 15 with congenital limb loss (44%). Athletes age 20–29 years experienced most injuries, four per athlete. The glenohumeral joint was the reported injury site (23%, *n* = 38). Index (first) injuries accounted for 77% (*n* = 128) injuries, 17% (*n* = 28) a recurrence and 6% (*n* = 10) an exacerbation. More than half of injuries occurred in training (58%, *n* = 94), this being slightly higher in those with traumatic limb loss. Athletes with quadruple levels of limb deficiency had double the number of recurrent injuries as those with single or double limb deficiency.

**Conclusion:**

Elite athletes with limb deficiency experience upper quadrant injuries, with glenohumeral joint the most frequently reported. The quality and consistency of data reported limits definitive conclusions, although findings highlight the importance of precision and accuracy in recording injury surveillance to enable implementation of effective injury prevention strategies.

## Background

Paralympic sport participation has grown considerably since the first Stoke Mandeville Games in 1948, with over 4000 athletes taking part in the London 2012 Paralympic Games [1]. Despite this growth, injury surveillance data within this population remains scarce. Injury surveillance is vital to understand the aetiology and prevalence of common injuries within specific sporting populations so that effective, injury prevention strategies can be developed [2]. Existing research from Paralympic populations evidences poor methodological quality, inconsistent injury definitions and heterogeneity across studies, making it difficult to draw definitive conclusions [3]. There is extensive literature regarding injury surveillance and injury prevention programmes in able-bodied athletes [4–6] resulting in reduced healthcare costs and reduced rehabilitation time post injury [7]. Significant opportunities now exist to extend this to other elite sporting populations.

Data suggests that shoulder injuries account for the majority of injuries for athletes with physical impairments, with more than 31 lost days to training over a 3-year period, compared with 17 for all other injury sites [7]. Shoulder injuries also account for the highest percentage (25%) of ‘major injuries’, defined as ‘22 or more days lost due to injury’, compared with all other body areas (19%) [8]. Amputees, or as classified by the International Paralympic Committee, individuals with ‘Total or partial absence of bones or joints as a consequence of trauma (e.g. car accident), illness (e.g. bone cancer) or congenital limb deficiency (e.g. dysmelia)’ [9]are just one of the eligible impairment groups within Paralympic Sport [9] and are at risk of developing upper limb injuries due to their unique biomechanical abnormalities [10–12]. From evidence in amputees with lower limb deficiency, strength discrepancies between the residual and contralateral limb exist thus disrupting the kinetic chain [10, 11]. In amputees with upper limb deficiency, compensation for the loss of movement and function in the missing limb, heighten functional demand and workload on the contralateral arm, increases the potential for musculoskeletal injury [12, 13].

Recognising the physical, physiological and biomechanical impact of limb deficiency in Paralympic athletes, injury surveillance data, including detail of aetiological factors is required across the upper quadrant to inform the development of proactive strategies to mitigate the risk of injury and subsequent impact of injuries on sporting performance [3]. For athletes with limb deficiency it may be useful to consider the involvement of the trunk and more specifically the thorax, including the thoracic spine; centrally located within functional kinetic chains. In a trial involving elite handball players (*n* = 660) a lower prevalence of shoulder problems were recorded across a season for those who completed an injury prevention programme which included thoracic mobility exercises [14]. Within Paralympic sport we first need to understand what the nature, scope and burden of injuries are to inform further research.

The aim of this study is therefore to investigate injuries of the upper quadrant in elite athletes with limb deficiency. Key objectives include:

1. To Identify upper quadrant injury frequency in elite athlete with limb deficiency, including recurrence and exacerbation
2. To explore clinical findings (aetiological factors and clinical examination findings) of elite athletes with limb deficiency presenting with upper quadrant injuries
3. To examine the conservative injury management and onward referral of elite athletes with limb deficiency.

## Methods

### Design

A retrospective analysis of data collected from a cohort of elite athletes with limb deficiency captured from medical records (physiotherapy and medicine) extracted from The English Institute of Sport (EIS) Injury & Illness Performance Project using their online notes systems; ‘Performance Data Management System’ (PDMS) and ‘I-Zone’.

### Inclusion criteria

All ‘elite’ athletes with limb deficiency, treated within an EIS or relevant National Governing Body setting between January 2008 and February 2016 who had, in line with usual practice in elite sport in the United Kingdom, self-referred to a physiotherapist with an upper quadrant injury, defined as ‘tissue damage or other derangement of normal physical function due to participation in sports, resulting from rapid or repetitive transfer of kinetic energy’ [15] were selected if they met inclusion criteria:

- National Governing Body funded to either ‘Podium’ or ‘Podium Potential’ level; therefore, deemed ‘elite’ and eligible for physiotherapy support within an EIS setting, by an EIS or National Governing Body practitioner.
- Having a limb deficiency, either upper and/or lower limb.
- All levels of limb deficiency, including part of the hand or foot.

### Injury definition

All injury records extracted from the database were classified according to the following descriptions and based on existing classification [16] where stage of recovery differentiates ‘exacerbation’ from ‘reoccurrence’. For the purpose of this study 6 months was deemed an appropriate cut off based on tissue healing:

1. Index injury: first presentation to a physiotherapist with a complaint of an upper quadrant complaint [cervical spine, thorax (thoracic spine and ribs)], shoulder, upper arm, elbow, forearm, wrist and hand.
2. Injury exacerbation: an injury of the same type, at the same site as an index injury, occurring < 6 months after the index injury.
3. Injury reoccurrence: an injury of the same type and site as an index injury, occurring > 6 months after the index injury

### Data collection

Data on injuries acquired ‘directly’ or ‘indirectly’ from participation in sport [15] were extracted from ‘I-Zone’, in the form of physiotherapy management documentation (2008 to 2015) including athlete musculoskeletal injuries in the years immediately preceding inception of ‘I-Zone’, and ‘Performance Data Management System’ (2015 to 2016). In the absence of an established approach to injury surveillance, data relating to the following were extracted from medical notes (UK legal requirement) where available: mechanism of injury, classification according to body region and structure (e.g. joint), aetiological factors derived from patient history (e.g. fall), and clinical findings according to physical examination (e.g. muscular weakness, joint stiffness), clinical and medical management including number of treatments per injury and onward referral for investigations.

These data were stored on an encrypted coded secured hard drive. This data was kept secure by an external EIS administrator in a password protected file. Data was anonymised and individual sports removed before being provided to the lead researcher (LH) by the EIS PDMS management team (PM). Data, including recorded instances of injuries sustained that pre-dated 2008, was extracted based primarily on location of injury with impairment and limb deficiency (congenital or traumatic) documented to enable analysis of discrete groups.

### Ethical approval

Ethical approval was granted by the School of Sport, Exercise and Rehabilitation Sciences Ethics Committee, University of Birmingham. Participants had given written consent for their medical records to be used for the purpose of research on admission to their sport’s world class programme. Participants were assigned a unique identifier code to assure their identity was protected and anonymity assured. All sport-related identifiable data was removed. Data were extracted, where available, according to the aims and objectives e.g. nature and location of injury, as well timing, investigations, management.

### Data analysis

Descriptive analysis was performed on athlete demographics, disability characteristics, injury location, injury characteristics, clinical findings, conservative management and onward referral using mean, range, frequencies and percentages as appropriate. Histograms were used to visually display results and to enable examination across groups, and according to level of limb deficiency. All data analysis was performed using SPSS version 23.

### Patient and public involvement

The study was conceived from our working with elite athletes with limb deficiency over many years. Study findings will be disseminated to practitioners at the English Institute of Sport, and athletes and families via conference presentations, newsletters and through social media.

## Results

### Participant characteristics

Data are included from 34 athletes with limb deficiency (see Table 1), with the majority having single limb deficiency (*n* = 25), seven double limb deficiency and two quadruple limb deficiency. Sports included Powerlifting, Para-Archery, Wheelchair Basketball, Para-Cycling, Para-Canoe, Para-Triathlon, Para-Sailing, Para-Shooting, and Para-Swimming. Participants presented with congenital limb loss (44%, *n* = 15) e.g. dysmelia, and traumatic limb loss (56%, *n* = 19) including elective amputations (see Table 1). The mean age at injury onset was 28 (range 16–50) years, with this being higher in those with traumatic limb loss compared to congenital limb loss, 29 (16–50) and 26 (16–42) years respectively.

**Table 1:** Participant characteristics

Impairment	Female: Male Athletes (*n*=)	Congenital limb loss (*n*=)	Traumatic limb loss (*n*=)	Total injuries (*n*=)
Single Trans Tibial Amputee (A)	2:3	1	4	**34**
Bilateral Trans Tibial Amputee (B)	0:1	0	1	**34**
Single Trans Femoral Amputee (C)	4:5	1	8	**16**
Bilateral Trans Femoral Amputee (D)	1:0	1	0	**4**
Single Through Knee Amputee (E)	0:2	0	2	**6**
Trans Femoral Amputee & Through Knee Amputee (F)	0:1	0	1	**7**
Trans Tibial Amputee & Through Knee Amputee (G)	0:1	1	0	**4**
Trans Tibial Amputee & Upper Limb Loss (H)	0:1	1	0	**18**
Trans Femoral Amputee & Upper Limb Loss (I)	1:1	1	1	**2**
Above Elbow Amputee (J)	1:1	1	1	**2**
Below Elbow Amputee (K)	2:2	3	1	**21**
Bilateral Upper Limb Loss (L)	1:1	2	0	**3**
Unilateral Hand Loss (M)	2:1	3	0	**11**
Total	**14:20**	**15 (44%)**	**19 (56%)**	**162**

### Characteristics of injuries

**Fig. 1:**
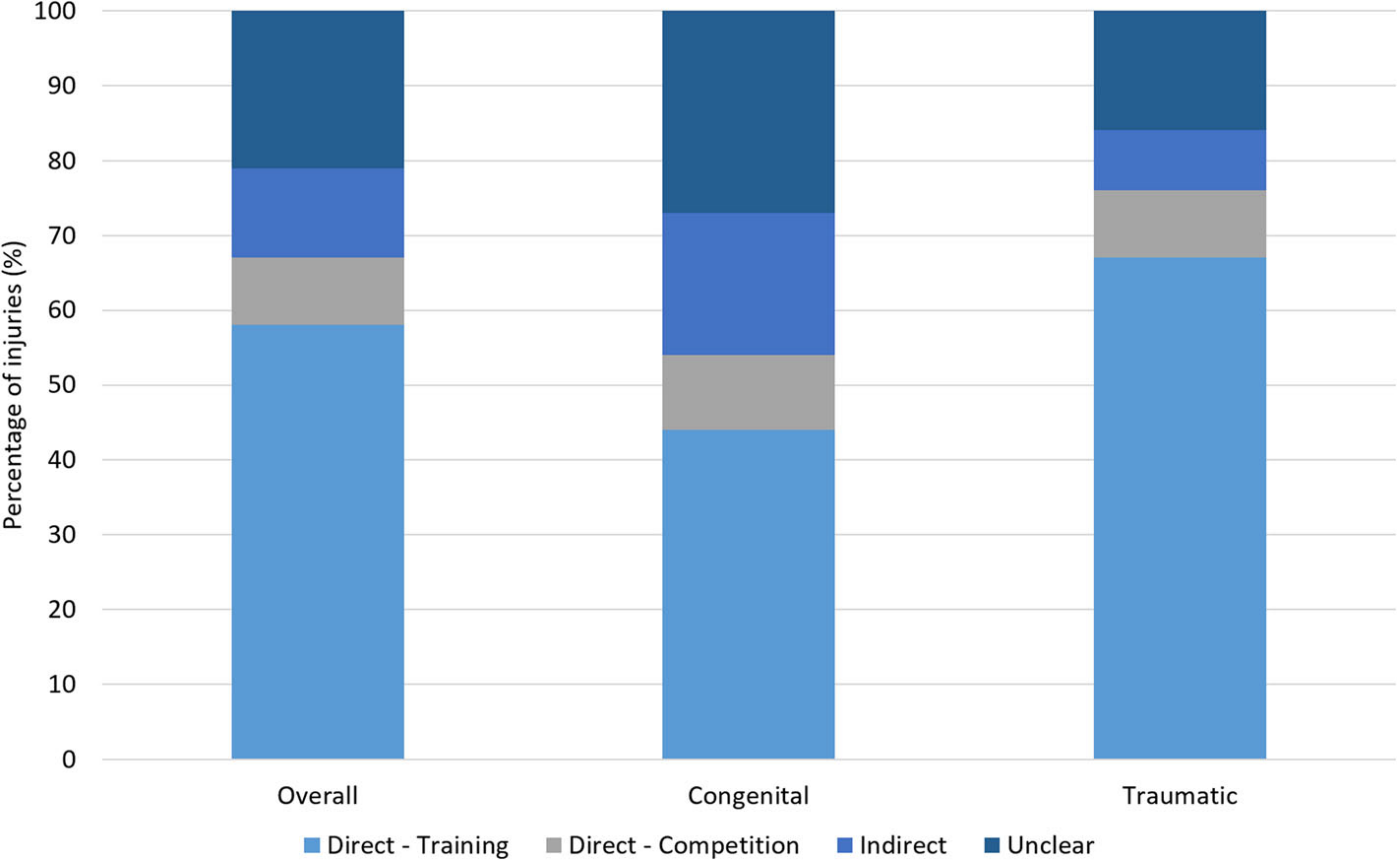
Injury occurrence: congenital and traumatic limb loss

A total 162 injuries were recorded, see Table 2. Of these, 77% (*n* = 124) were index (first) injuries, 17% (*n* = 28) were a recurrence and 6% (*n* = 10) an exacerbation. More than half the injuries occurred directly from training (58%, *n* = 94) or competition (9%, *n* = 15), a small number indirectly (12%, *n* = 20) and many reporting onset as ‘unclear’ (21%, *n* = 33). Across disability groups a higher relative percentage of injuries occurred during training in the traumatic compared to congenital limb loss group, 67% (*n* = 66) and 44% (*n* = 28) respectively. Although for the congenital limb loss group there was less clarity regarding timing, with 27% (*n* = 17) ‘unclear’ compared to 16% (*n* = 16) in the trauma group (Fig. 1). With respect to age groups, athletes in the 20–29 age range experienced more injuries than other age groups with 4 injuries per athlete (*n* = 18).

**Table 2:** Frequency of injuries according to limb deficiency

	Disability Group													
Location of Injury (*n*=)	A	B	C	D	E	F	G	H	I	J	K	L	M	Total injuries
Glenohumeral	13	5	1	0	2	1	1	7	0	0	4	0	4	**38**
Cervical	5	3	4	0	2	3	0	4	0	0	5	2	2	**30**
Thorax	3	5	3	0	1	2	1	0	0	0	8	0	2	**25**
Elbow	10	6	2	2	0	0	0	2	0	0	1	1	1	**25**
Neck and shoulder	0	7	1	0	0	0	0	1	1	0	1	0	0	**11**
Non-specific shoulder	1	2	2	1	0	0	0	2	0	1	0	0	0	**9**
Upper arm	0	2	1	0	1	0	1	0	1	1	0	0	0	**7**
ACJ/ Clavicle	0	0	0	1	0	0	0	0	0	0	2	0	1	**4**
Forearm	0	1	2	0	0	0	0	0	0	0	0	0	0	**3**
Wrist	0	0	0	0	0	1	1	0	0	0	0	0	0	**2**
Hand/fingers	1	0	0	0	0	0	0	1	0	0	0	0	0	**2**
Neural	0	0	0	0	0	0	0	1	0	0	0	0	0	**1**
Other	1	3	0	0	0	0	0	0	0	0	0	0	1	**5**
Total injuries	**34**	**34**	**16**	**4**	**6**	**7**	**4**	**18**	**2**	**2**	**21**	**3**	**11**	**162**

Abbreviations: *A* Single trans tibial amputee. *B* Bilateral trans tibial amputee. *C* Single trans fermoral amputee. *D* Bilateral trans femoral amputee. *E* Single through knee amputee. *F* Trans femoral amputee and through knee amputee. *G* Trans tibial amputee and through knee amputee. *H* Trans tibial and upper limb loss. *I* Trans femoral and upper limb loss. *J* Above elbow amputee. *K* Below elbow amputee. *L* Bilateral upper limb loss. *M* = Unilateral Hand Loss. *ACJ* = acromioclavicular joint

### Frequency of injuries

**Fig. 2:**
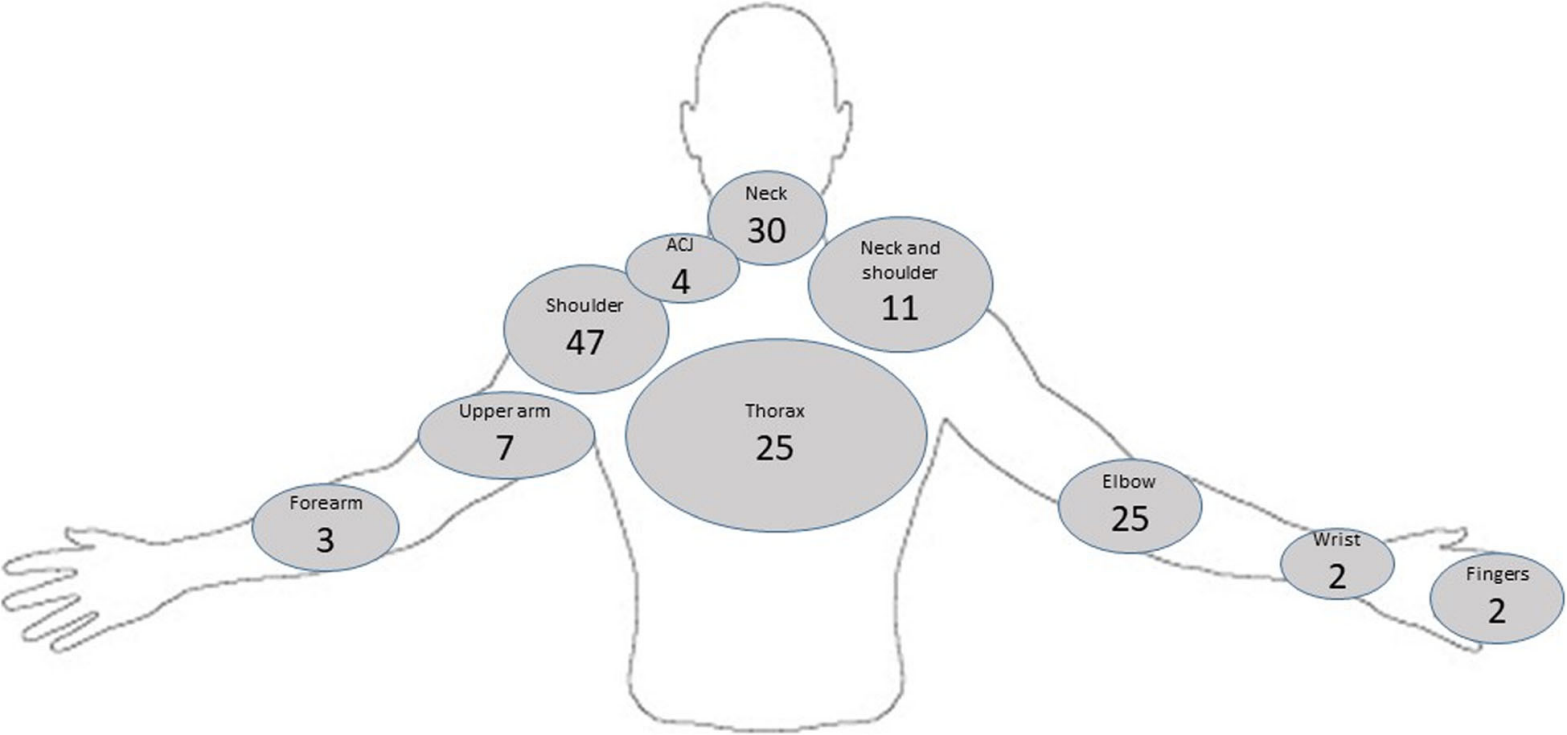
Injury frequency

The number of injuries reported by athletes varied considerably from 1 injury through to 13 injuries, with 7 athletes experiencing 2 injuries and most (*n* = 20) experiencing fewer than five injuries. The frequency of injuries with respect to location across disability groups are reported in Table 2, with Fig. 2 illustrating frequency according to upper quadrant body regions. The glenohumeral joint was the most commonly recorded injured site (23%, *n* = 38), although when combined with ‘non-specific shoulder’ (6%, *n* = 9) this accounts for more than a quarter of documented injuries (29%, *n* = 47). For glenohumeral joint injuries, no differences were seen between congenital and traumatic limb loss groups, 23% (*n* = 15) and 24% (*n* = 23) respectively and this the most common injury site for both groups. Glenohumeral joint injuries were also the most common site of injury for single level, 24% (*n* = 27), and multi-level amputees at 39% (*n* = 7).

**Fig. 3:**
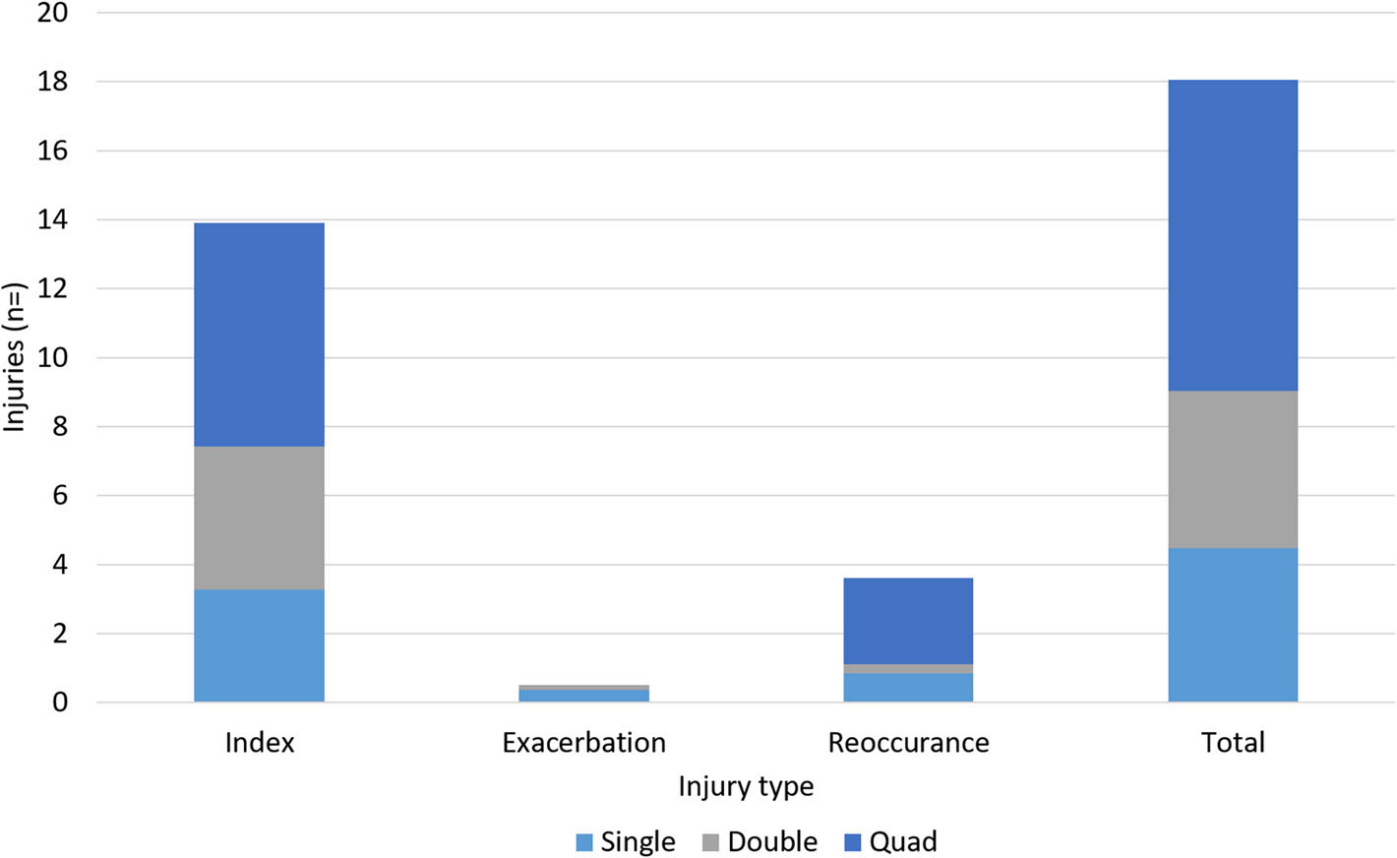
Number of injuries per athlete according to limb loss categories

Differences in the number of injuries per athlete according to limb deficiency are presented in Fig. 3. Athletes with quadruple limb deficiency (*n* = 2) had double the number of injuries compared to those with single level of limb deficiency Injury reoccurrence appears to be higher in this group too. Patterns of injury occurrence (index, exacerbation, recurrent) were comparable across athletes with congenital or traumatic limb loss.

### Clinical findings

**Fig. 4:**
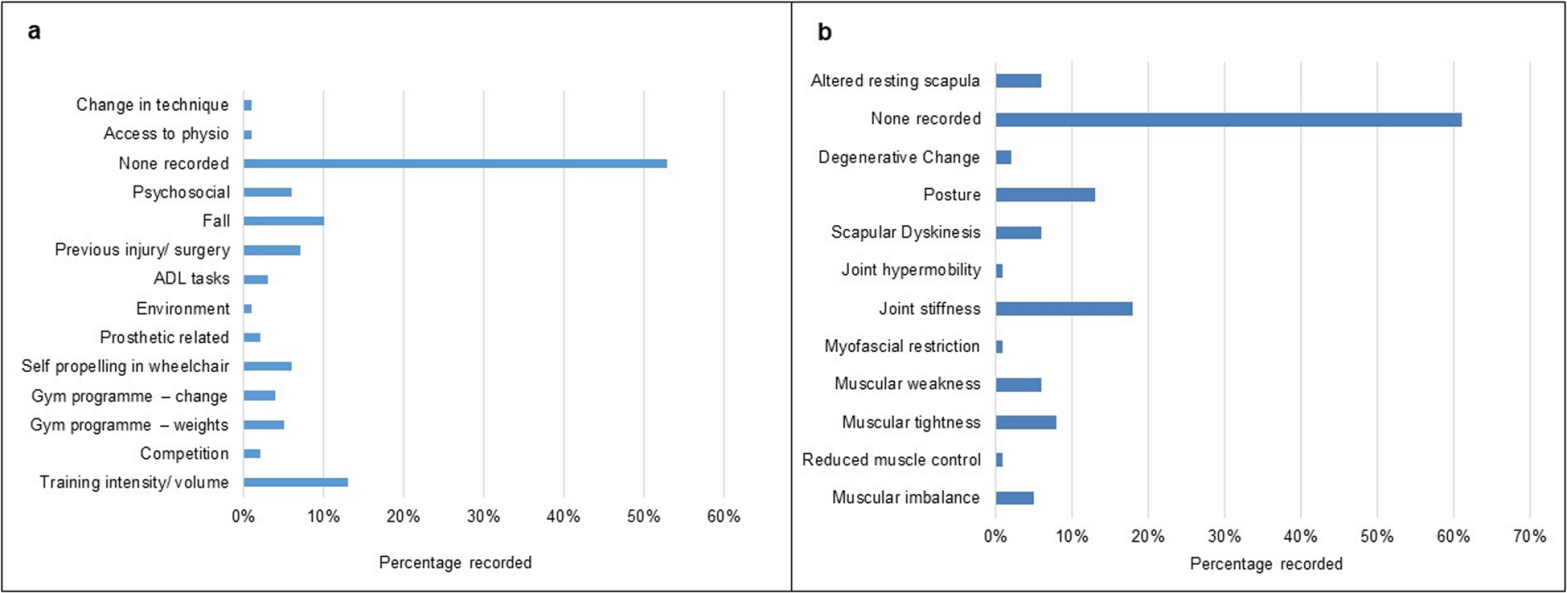
**a**, **b**. Patient reported aetiological factors and therapist reported clinical findings

Patient reported aetiological factors and therapist reported clinical findings were explored in relation to injury onset (Fig. 4a and b) across the whole sample. Notwithstanding the paucity of detail, training volume or intensity was reported most frequently (13%, *n* = 21), followed by a fall (10%, *n* = 16). In terms of falls, 69% were in athletes with lower limb deficiency (*n* = 11) and 31% in those with upper limb deficiency (*n* = 5). In terms of physiotherapist findings on examination, joint stiffness was reported most frequently (18%, *n* = 29), followed by posture (13%, *n* = 25).

### Conservative injury management, onward referral and outcome

**Fig. 5:**
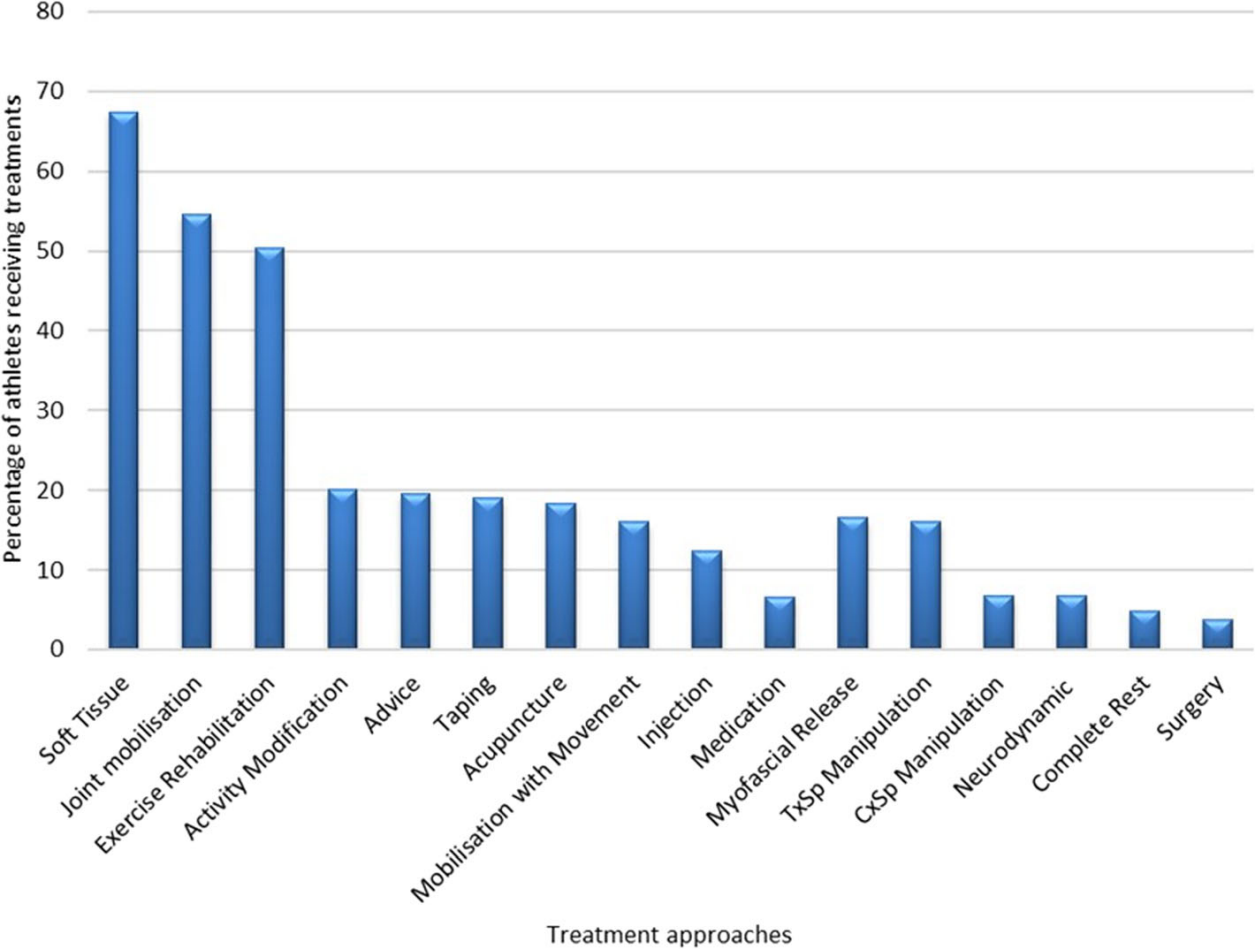
Management

Injury management, including physiotherapy and medical interventions, was evaluated to examine frequency of approaches used in athletes (Fig. 5). More than half of the athletes received soft tissue techniques, joint mobilisation and exercise rehabilitation, with documentation suggesting that less than a quarter received activity modification and advice. Injection therapy was used in 10% of athletes, with complete rest or surgery was reported in < 5% of athletes.

Number of treatments for injuries varied considerable from one (26%, *n* = 43), two (19%, *n* = 31), three (14%, *n* = 22), four (11%, *n* = 18) right through to 43 treatments in one instance. Across all injuries, the mean number of treatments was 4.8. Of all the injuries seen, 35% (*n* = 58) were referred on for further medical investigation. Ultrasound was the most frequently used modality (18%, *n* = 29), followed by MRI (10%, *n* = 16), and X-ray (6%, *n* = 10).

Evidence to suggest injury resolution had been achieved on discharge was unclear in 66% (*n* = 108) of injuries. Additionally with 19% (*n* = 31) records stating ‘open for review’ the outcome was also unclear. Just 9% (*n* = 14) of reported injuries reported full resolution with 3% (*n* = 4) being referred on to doctor.

### Missing data

Of all 162 injury records for the 34 athletes, records had data missing. These included missing notes within physiotherapy documentation (*n* = 16), no record of physiotherapy assessment and management despite referral (*n* = 11) and details of medical investigation (*n* = 10).

## Discussion

This is the first report of injury surveillance of elite para-athletes with limb deficiency. Irrespective of location and level of limb deficiency, injuries to the glenohumeral joint were the most frequently reported in this population of elite athletes with limb deficiency. This is in line with previous research [4] regarding shoulder injuries in athletes with various physical impairments.

### Frequency of injury

Athletes with single and double trans-tibial limb deficiency reported glenohumeral joint, neck and shoulder and elbow joint injuries. This is perhaps not surprising given maximal strength and power needed for optimising overhead performance is dependent on the transmission of kinetic energy, created in the lower limbs, to the shoulder via the pelvis [17]. According to Kibler [18], the shoulder is central to the kinetic chain, through transference of force from the lower limbs to the hand via the trunk. In athletes with lower limb deficiency, this is disrupted and results in significant strength discrepancies between the residual and contralateral limb [10, 11].

Where fewer injuries were reported in athletes with transfemoral limb deficiency this may be a consequence of participation in wheelchair rather than ambulant sports, and there being a protective effect of the equipment for the upper quadrant, contributing to fewer shoulder injuries [19]. Athletes with upper limb deficiency present with spinal asymmetries, lateral shift, scoliosis, and shoulder elevation on the side of limb deficiency [20], potentially contributing to injuries in this region. Disruption to the kinetic chain in athletes with lower limb deficiency could result in an increase in forces being transmitted through the thorax resulting in musculoskeletal injury [21].

Neck and shoulder were most the most frequently reported site in relation to exacerbation. For some, this could be a consequence of wheelchair propulsion [22]. Recovery from neck and shoulder injuries may require rest from sporting activity however, wheelchair dependant athletes will require their upper limbs for activities of daily living e.g. transfers, which may account for the increased numbers of injury exacerbations and reoccurrences.

There are many variations and inconsistencies of injury types within injury surveillance literature [16] making comparison difficult. Early consensus statements advocated that injury types are based on return-to-play criteria allowing for improved reporting consistency and comparisons to be made across sporting populations [23, 24]. The most recent consensus statement from the International Olympic Committee (IOC) details a robust methodological framework to support comprehensive recording and reporting of epidemiological data on injuries [15] which is needed to improve injury surveillance in Paralympic populations.

### Clinical findings

From the athlete history, a recent increase in training volume or intensity was reported as a contributing factor. There is no research investigating training workloads and injury onset in a Paralympic population yet it has been reported that an increase in acute training loads can be a predictor of injury in able-bodied athletes [25], particularly subjective workloads [26]. In the absence of more detailed internal and external training load data and a lack of understanding regarding the impact of unique individual biomechanics, definitive conclusions cannot be drawn. Finding from this study infer that athletes with traumatic limb deficiency may be more susceptible to training-related injuries. This may be a consequence of taking up sport at a later age, compared with athletes with congenital limb deficiency, and therefore demonstrate a reduced chronic workload.

Falls were also reported, particularly in athletes with lower limb deficiency, and supported by previous research in a non-athletic limb deficiency population [27]. The authors are not aware of research investigating the cause of falls in an athletic population with limb deficiency. It is feasible that an athletic population participates in higher level activities compared with a non-athletic population thus increasing their risk of falls. The contribution of equipment, such as prosthetics to falls is unknown with only 2% reporting this as a contributing factor to injury onset.

Joint stiffness was the largest clinical finding for injury presentation followed by posture, and scapular dyskinesia. It is documented in the literature that there is an association between altered scapula kinematics and upper quadrant pathology [25, 28] which could have contributed to the high levels of injuries reported within the shoulder complex. Whilst relatively under researched the relationship between the thoracic spine and upper quadrant has recently gained some momentum, with evidence of a kinematic relationship between mobility in the thoracic spine and shoulder [29] and neck [30]. With a recent review synthesising evidence of thoracic spine exercises for mobility, motor control, work capacity and strength, there now exists a clinical reasoning framework to support personalised exercise prescription and rehabilitation for athletes with impairments [31].

### Conservative injury management and onward referral

Injury diagnosis in the majority of cases was based on clinical assessment by a physiotherapist, supported in some cases by a doctor, rather than medical investigation. Clinical diagnosis may vary between and within professions [32] and is in part illustrated here with the number of different terms used to suggest an injury if the shoulder region. Diagnoses such ‘non-specific shoulder injury’ may have an unclear diagnosis, and thus account for the increased number of injury recurrences and exacerbations. In the absence of a clear clinical diagnosis along with etiological factors contributing to a clinical complaint, management is likely to be less effective and recovery may take longer [33].

Where just 50% of athletes received exercise rehabilitation, this was likely a consequence of collaborative and multidisciplinary management involving strength and conditioning coaches. Where these data were not recorded by the physiotherapists, caution should be taken in drawing definitive conclusions regarding scope of injury management and in particular the use of exercise within rehabilitation.

In this study, it was unclear on termination of treatment whether the injury had fully resolved and if the athlete had successfully returned to play. This limits the accuracy of the results as the level of sporting activity that the athlete returned to and when, remains unclear [16]. As a result, we defined injury recurrence as occurring more than 6 months after the onset of the index injury, proposing that at this stage of tissue healing, injuries would be in the remodelling phase and therefore athletes are likely to have returned to play.

### Strengths and limitations

Data was drawn from all elite athletes with limb deficiency during a 12-year period. Despite the relatively small sample, important findings regarding injury frequency across different groups with limb deficiency provide a foundation for further research. Where the researcher was blinded to each individual sport, researcher bias was minimised. Blinding to individual sports was on the contrary a significant limitation and precluded evaluation of injuries for specific sports. To draw valid conclusions and make recommendations for injury prevention strategies for specific sports this information would be useful. Information regarding wheelchair dependency would also give an insight into possible risk factors for this population and a deeper insight into protective effects from shoulder injury e.g. disruption across the kinetic chain.

Poor reporting and lack of standardisation precluded the assessment of injury severity, previously defined by Fuller in 2006 as, ‘the number of days from date of injury to the date of return to full participation in training, and availability for match selection’ [16]. We are therefore unable to compare current findings with previous research of athletes with disabilities [4]. Additionally and in line with recently published guidelines [15] data to accurately report time for return to play following injury was not possible.

Data were taken from medical notes that lacked sufficient detail, with over 50% of injuries providing no aetiological data or clinical findings for analysis, including 34 data sets with missing information. The main reason for missing data was treatment by a practitioner who did not have access to either of the electronic medical record systems, ‘PDMS’ and ‘I-Zone’. In addition, inconsistencies in terminology used between clinicians, and diagnoses based on clinical assessment at this time may have influenced the number of specific injuries recorded.

### Practice and research recommendations

Consistent use of language, terminology and accurate medical records are required for detailed injury surveillance and the development of effective strategies to mitigate the threat of injury in Paralympic sport. The adoption of IOC Consensus Statement [15] would enhance the consistency and quality of data used to underpin preventative approaches directly relevant and accessible to practitioners and athletes with limb deficiency. As Finch states, ‘standardised injury data collection is crucial to underpin the provision of safe opportunities for all those who participate in sport’ [34] and this is no different for athletes with physical impairments.

## Conclusion

Elite athletes with limb deficiency experience upper quadrant injuries, with glenohumeral joint the most frequently reported, and comparable across congenital and traumatic limb deficient groups. Findings highlight the importance of injury surveillance in athletes with limb deficiency to enable implementation of effective injury prevention strategies. Results suggest specifically targeting the high levels of injuries recorded in the region of the glenohumeral joint/shoulder, including further research to determine involvement or disruption across the kinetic chain.

## Data Availability

The data is derived from patients notes and given the nature of the sample and characteristics is not available for view. Queries regarding the data can be directed to the corresponding author n.heneghan@bham.ac.uk

## Abbreviations

ACJ: Acromioclavicular joint
ADL: Activities of daily living
CxSp: Cervical spine
EIS: English Institute of Sport
IOC: International Olympic Committee
IPC: International Paralympic Committee
PDMS: Performance Data Management System
TxSp: Thoracic spine

## References

[1] Webborn N, Emery C (2014). Descriptive epidemiology of Paralympic sports injuries. PM R.

[2] Finch C (2006). A new framework for research leading to sports injury prevention. J Sci Med Sport.

[3] Weiler R (2016). Sport injuries sustained by athletes with disability: a systematic review. Sports Med.

[4] Ferrara MS, Peterson CL (2000). Injuries to athletes with disabilities: identifying injury patterns. Sports Med.

[5] Myklebust G, Engebretsen L, Braekken IH, Skjølberg A, Olsen OE, Bahr R (2003). Prevention of anterior cruciate ligament injuries in female team handball players: a prospective intervention study over three seasons. Clin J Sport Med.

[6] Gagnier JJ, Morgenstern H, Chess L (2013). Interventions designed to prevent anterior cruciate ligament injuries in adolescents and adults: a systematic review and meta-analysis. Am J Sports Med.

[7] Hewett TE, Ford KR, Myer GD (2006). Anterior cruciate ligament injuries in female athletes: part 2, a meta-analysis of neuromuscular interventions aimed at injury prevention. Am J Sports Med.

[8] Ferrera MS and Buckley WE, *Athletes With Disabilities Injury Registry* Adapted Physical Activity Quarterly, 1996(13): p. 50–60.

[9] Committee, I.P. Classification explained. 2020; Available from: https://www.paralympic.org/classification.

[10] Nolan L, Wit A, Dudziñski K, Lees A, Lake M, Wychowañski M (2003). Adjustments in gait symmetry with walking speed in trans-femoral and trans-tibial amputees. Gait Posture.

[11] Prinsen EC, Nederhand MJ, Rietman JS (2011). Adaptation strategies of the lower extremities of patients with a Transtibial or Transfemoral amputation during level walking: a systematic review. Phys Med Rehabil.

[12] Jones LE, Davidson JH (1999). Save that arm: a study of problems in the remaining arm of unilateral upper limb amputees. Prosthetics Orthot Int.

[13] Ostlie K (2011). Musculoskeletal pain and overuse syndromes in adult acquired major upper-limb amputees. Arch Phys Med Rehabil.

[14] Andersson S (2017). Preventing overuse shoulder injuries among throwing athletes: a cluster randomised controlled trial in 660 elite handball players. Br J Sports Med.

[15] Bahr R, Clarsen B, Derman W, Dvorak J, Emery CA, Finch CF, Hägglund M, Junge A, Kemp S, Khan KM, Marshall SW, Meeuwisse W, Mountjoy M, Orchard JW, Pluim B, Quarrie KL, Reider B, Schwellnus M, Soligard T, Stokes KA, Timpka T, Verhagen E, Bindra A, Budgett R, Engebretsen L, Erdener U, Chamari K (2020). International Olympic Committee consensus statement: methods for recording and reporting of epidemiological data on injury and illness in sport 2020 including STROBE Extension for Sport Injury and Illness Surveillance (STROBE-SIIS). Br J Sports Med.

[16] Fuller CW (2007). A framework for recording recurrences, reinjuries, and exacerbations in injury surveillance. Clin J Sport Med.

[17] Manske R, Ellenbecker T (2013). Current concepts in shoulder examination of the overhead athlete. Int J Sports Phys Ther.

[18] Kibler WB, Safran MR (2000). Musculoskeletal injuries in the young tennis player. Clin Sports Med.

[19] Fullerton HD, Borckardt JJ, Alfano AP (2003). Shoulder pain: a comparison of wheelchair athletes and nonathletic wheelchair users. Med Sci Sports Exerc.

[20] Greitemann B, Güth V, Baumgartner R (1996). Asymmetry of posture and truncal musculature following unilateral arm amputation: a clinical, electromyographic, posture analytical and photogrammetric study. J Orthop.

[21] Kibler WB, Press J, Sciascia A (2006). The role of core stability in athletic function. Sports Med.

[22] Mercer JL (2006). Shoulder joint kinetics and pathology in manual wheelchair users. Clin Biomech.

[23] Fuller CW (2006). Consensus statement on injury definitions and data collection procedures in studies of football (soccer) injuries. Clin J Sport Med.

[24] Fuller CW (2007). Consensus statement on injury definitions and data collection procedures for studies of injuries in rugby union. Br J Sports Med.

[25] Kibler WB (2013). Clinical implications of scapular dyskinesis in shoulder injury: the 2013 consensus statement from the ‘scapular summit’. Br J Sports Med.

[26] Saw AE, Main LC, Gastin PB (2016). Monitoring the athlete training response: subjective self-reported measures trump commonly used objective measures: a systematic review. Br J Sports Med.

[27] Kulkarni J (1996). Falls in patients with lower limb amputations: prevalence and contributing factors. Physiotherapy.

[28] Cools AM, Struyf F, De Mey K, Maenhout A, Castelein B, Cagnie B (2014). Rehabilitation of scapular dyskinesis: from the office worker to the elite overhead athlete. Br J Sports Med.

[29] Heneghan NR, Webb K, Mahoney T, Rushton A (2019). Thoracic spine mobility, an essential link in upper limb kinetic chains in athletes: a systematic review. Transl Sports Med.

[30] Tsang SM, Szeto GP, Lee RY (2013). Normal kinematics of the neck: the interplay between the cervical and thoracic spines. Man Ther.

[31] Heneghan NR, Lokhaug SM, Tyros I, Longvastøl S, Rushton A. A clinical reasoning framework for thoracic spine exercise prescription in sport: a systematic review and narrative synthesis. BMJ Open SEM. 2020;6:e000713. 10.1136/bmjsem-2019-000713 PMC7173996 32341799

[32] Spoto MM, Collins J (2008). Physiotherapy diagnosis in clinical practice: a survey of orthopaedic certified specialists in the USA. Physiother Res Int.

[33] Doody C, McAteer M (2002). Clinical reasoning of expert and novice physiotherapists in an outpatient Orthopaedic setting. Physiotherapy.

[34] Finch C, Staines C (2018). Guidance for sports injury surveillance: the 20-year influence of the Australian sports injury data dictionary. Inj Prev.

